# Risk factors and scores for prediction of coronary artery aneurysms in Kawasaki disease: a European monocentric study

**DOI:** 10.1186/s12887-024-04623-3

**Published:** 2024-02-23

**Authors:** Adriano La Vecchia, Rita Stracquadaino, Lucia Mauri, Lucia Augusta Baselli, Rozan Abdallah, Martina Cucchetti, Anna Maria Colli, Carlo Agostoni, Rosa Maria Dellepiane

**Affiliations:** 1 Department of Clinical Sciences and Community Health (DISCCO), University of Milan, Milan, Italy; 2 Department of Medicine and Surgery, University of Milan-Bicocca, Monza, Italy; 3 Pediatric Area, Fondazione IRCCS Ca’ Granda Ospedale Maggiore Policlinico, via della Commenda 9, Milan, 20122 Italy; 4 Department of Cardiology, Fondazione IRCCS Ca’ Granda Ospedale Maggiore Policlinico, Milan, Italy

**Keywords:** Kawasaki disease, Aneurysm, Risk scores, Risk factors, Outcome, Cardiology, Children

## Abstract

**Background:**

Japanese Kawasaki disease (KD) risk scores cannot be adopted in non-Japanese patients. In North American populations a baseline coronary artery Z-score > 2 and the Son score are associated with coronary artery aneurysms (CAAs) at 4 and 8 weeks from disease onset. In European populations, the Kawanet and Kawanet-echo scores are associated with intravenous immunoglobulin resistance. This study aims to evaluate the association between KD risk scores and baseline coronary artery Z-scores with CAAs at one, two, and six months in a European population.

**Methods:**

Historical cohort study of all the children diagnosed with KD in a tertiary care hospital in Milan, Italy, between 1st January 2015 and 31st May 2021. Univariate and multivariate (adjusting for age and corticosteroid therapy) logistic regression analyses were used to study the association between the risk scores, a baseline Z-score ≥ 2 and ≥ 2.5 with CAAs.

**Results:**

Eighty-nine patients were diagnosed with KD at our Centre, and 12 were excluded based on the exclusion criteria. We included 77 patients, 51 (66%) males, and 26 (34%) females, with a median age at presentation of 27 months (IQR 13–46). A baseline Z-score ≥ 2 was correlated with CAAs at one and two-month follow-ups (odds ratio (OR) 10, 95% confidence interval (CI) 2–72, and OR 18, CI 3-357) but not at six-month follow-up. The Son score showed an association with one and two-month follow-up CAAs (OR 3, CI 1.3-7, and OR 3, CI 1.3-8) but not with a six-month follow-up.

**Conclusions:**

Patients with a baseline Z-score ≥ 2 are at higher risk for CAAs in the long term. The Son score should be tested in larger European samples. Further studies should keep the observational periods longer than 8 weeks from KD onset.

## Background

Kawasaki disease (KD) is an acute self-limited vasculitis affecting medium-sized arteries with a predilection for coronary arteries. In Europe, incidence rates range from 4.5 to 9 per 100 000 children under 5, who represent almost 85% of affected patients [1]. Coronary artery aneurysms (CAAs) are a long-term consequence of KD that can lead to myocardial infarction, heart failure, or death. Without treatment, almost 25% of patients develop CAAs while treatment with intravenous immunoglobulin (IVIG) reduces their incidence to 3–5% [2, 3].

In Japan in 1991, the Harada risk score [4] was developed to recognize indications for treatment with IVIG but in 2014 a retrospective study using data from Cleveland Hospital concluded that it could be used for selecting USA children at high risk for CAA development, though not sufficiently specific for select initial treatment [5]. Around 2006 and 2007, three risk scores were created to predict IVIG resistance, called Egami, Kobayashi, and Sano [6–8]. . However, none of the aforementioned risk scores can be adopted systematically in non-Japanese patients [9]. In North American populations, Son et all found a high predictive of a baseline coronary artery Z-score ≥ 2 for later development of CAA [10] and in 2019 retrospectively developed a risk model for CAA in KD based on demographic, laboratory, and echocardiography findings [11]. The Kawanet score was developed in France in 2020 to predict IVIG resistance in non-Asian populations [12]. In 2022, a multicentre study in Italy and France developed the Kawanet-echo score, a combination of the Kawanet score with the baseline echocardiography findings, that showed a better performance for IVIG resistance prediction than the Kawanet score and a maximal Z-score ≥ 2 [13]. Table 1 shows the scoring systems evaluated in the present study.

**Table 1:** Kawasaki disease scoring systems analysed in the present study

Risk score	Year and country	Risk factors	Points	Risk classes
Harada [4]	1991, Japan	- WBC count > 12 × 103/mm3 - Platelet count < 35 × 104/mm3 - C-reactive protein > 3 mg/dl - Hematocrit < 35% - Albumin < 3.5 g/dL - Age < 12 months - Sex Male	1 1 1 1 1 1 1	Low risk (0–3) High risk (≥ 4)
Egami [6]	2006, Japan	- Age < 6 months - Days of illness < 4 - Platelet count < 300 × 109/L - C-reactive protein > 8 mg/dL - ALT > 80 IU/L	1 1 1 1 2	Low risk (0–2) High risk (≥ 3)
Kobayashi [7]	2006, Japan	- Sodium ≤ 133 mmol/L - Days of illness at initial treatment ≤ 4 - AST ≥ 100 IU/L - Percentage of neutrophils ≥ 80% - C-reactive protein ≥ 10 mg/dL - Age ≤ 12 months - Platelet count ≤ 300 × 109/L	2 2 2 2 1 1 1	Low risk (0–3) High risk (≥ 4)
Sano [8]	2007, Japan	- C-reactive protein ≥ 7.0 mg/dL - Total bilirubin ≥ 0.9 mg/dL - AST ≥ 200 IU/L	1 1 1	Low risk (0–1) High risk (≥ 2)
Son [11]	2019, United States of America	- baseline Z-score of CA ≥ 2.0 - age < 6 months - Asian race - C-reactive protein ≥ 13 mg/dL	2 1 1 1	Low-Risk (0–1) Moderate risk (2) High risk (≥ 3)
Kawanet [12]	2020, France	- ALT > 30 IU/L - Hepatomegaly - Lymphocyte count < 2400/mm3 - Time to treatment < 5 days	1 1 1 1	Low risk (0–1) High risk (≥ 2)
Kawanet-echo score [13]	2022, Italy and France	- ALT > 30 IU/L - Hepatomegaly - Lymphocyte count < 2400/mm3 - Time to treatment < 5 days - Abnormal initial echocardiography defined as the presence at least 1 of the following: *CA maximal Z-score ≥ 2.0 *Pericarditis *Myocarditis *Ventricular dysfunction	1 1 1 1 2	Low risk (0–1) High risk (≥ 2)

ALT: Alanine aminotransferase

AST: Aspartate aminotransferase

CA: coronary artery

IU: international units

WBC: White blood cell count

The present investigation aims to assess the association of all these scores and of a baseline maximal coronary artery Z-score ≥ 2 and ≥ 2.5 with CAAs at one, two, and six months from the diagnosis in an Italian tertiary care Hospital.

## Materials and methods

### Design, setting, and patients

We performed a historical cohort study of patients diagnosed with KD in a tertiary care Hospital (IRCCS Fondazione Ca’ Granda Ospedale Maggiore Policlinico) in Milan, Northern Italy, between 1st January 2015 and 31st May 2021. Exclusion criteria were the second episode of KD, patients not treated with IVIG or treated after 10 days from the fever onset, no echocardiography data before the IVIG infusion or within 48 h after the IVIG infusion, not echocardiography data at one month nor two-month follow-up. The Milano Area 2 Ethical Committee approved the study, which included a waiver of informed consent because of the retrospective nature of the investigation (No 2018/0802).

### Treatment of the acute phase

All the patients included were treated with IVIG infusion at a dose of 2 g/Kg within 10 days from the fever onset. When the patients presented a resistant KD, defined as persistent or recrudescent fever after 36–48 h by the first IVIG infusion, a second IVIG infusion at the dose of 2 g/Kg was given as second treatment [3, 14, 15]. All the patients were treated with a high dose (30–50 mg/kg/day) of acetylsalicylic acid (ASA) until 48 h after disappearing of the fever, followed by a low dose (3–5 mg/kg/day) ASA continued depending on CAA development and size [14, 15]. Since the indications regarding corticosteroid therapy with IVIG and ASA were updated during the observational period [14], we adjusted our analyses for corticosteroid use as a possible confounding factor.

### Definitions

According to the American Heart Association (AHA) diagnostic criteria [16], we defined CAA as a maximal Z-score ≥ 2.5 either in the left or right coronary artery. The coronary artery Z-score was calculated based on Dallaire and Dahdah Z-scores [17]. Following the AHA criteria [16], complete KD was defined by the presence of prolonged unexplained fever together with at least 4 of 5 of the principal features, incomplete/atypical KD by prolonged unexplained fever with fewer than 4 principal clinical findings.

### Data collection and analysis

We collected data on demography (sex, birth date, and ethnicity), clinical symptoms (onset date defined as the first day the patient presented with signs related to KD, hospitalization date, clinical findings, and persisting or relapsing fever on day 2 after the initial IVIG therapy), therapy (treatment date, therapies used, therapeutic failure defined as the need of second-line therapy) and cardiac findings (date of echocardiography, coronary artery Z-scores, and other abnormalities as pericarditis, myocarditis and ventricular dysfunction). Data were collected from electronic records and patient charts.

We compared patients with baseline Z-score ≥ 2 and with patients with baseline Z-score < 2. The chi-square or Fisher’s exact tests were used for categorical variables and the Mann-Whitney U-test for continuous ones. Univariate and multivariate (adjusting for age and corticosteroid therapy) logistic regression analyses were used to derive the odds ratios (ORs) and 95% confidence intervals (CIs) between the risk scores, a baseline Z-score ≥ 2 and ≥ 2.5 with CAAs at one, two, and six-month follow-up. Statistical significance was considered as a *p*-value < 0.05. Statistical analysis was performed using R software (version 3.6.3 for Windows).

## Results

During the observational period, 89 patients were diagnosed with KD. We excluded 2 patients who did not receive the IVIG therapy, 8 patients who received IVIG therapy 10 days after the presentation, and 2 patients who had no data for the scores. We included 77 patients, 51 (66%) male, and 26 (34%) female, with a median age at presentation of 27 months (IQR 13–46). Table 2 gives the baseline characteristics, clinical data, and treatments of the patients, and the CAAs at one, two, and six months. Twelve (16%) patients needed a second IVIG infusion. Ten (13%) children received adjunctive steroid therapy with the first IVIG therapy, 6 (8%) of them by the intravenous route, and 4 (5%) by oral route, none of them repeated the steroid infusion. One (1%) patient received a steroid intravenous infusion with the second IVIG therapy. None of the patients received cyclosporine or biologic response modifiers.

**Table 2:** Baseline characteristics, clinical data and treatments of the patients and coronary artery aneurysms at one, two, and six months by baseline Z-score ≥ 2, <2 and total sample. Data are presented as median [interquartile range] or as frequency (percentage)

	Total sample, *n* = 77		Baseline Z-score ≥ 2, *n* = 26		Baseline Z-score < 2, *n* = 51		*p*-value
	MD	*n* (%)	MD	*n* (%)	MD	*n* (%)	
Age at disease onset in months, median [IQR]	0	27 [13–46]	0	14 [10–27]	0	34 [16–60]	< 0.01
Female	0	26 (34)	0	8 (31)	0	18 (35)	0.7
Ethnicity	2		1		1		1
European		60 (78)		21 (81)		39 (76)	
Asian		4 (5)		1 (4)		3 (6)	
Hispanic		5 (6)		1 (4)		4 (8)	
Arabic		5 (6)		2 (8)		3 (6)	
African		1 (1)		0		1 (2)	
Recent SARS-CoV-2 infection	0	5 (6)	0	1 (4)	0	4 (8)	0.7
Complete Kawasaki Disease	0	36 (47)	0	9 (35)	0	27 (53)	0.2
Days of symptoms at treatment, median [IQR]	1	7 [5–9]	0	8 [4–9]	1	7 [5–8]	0.7
Need for second infusion of IVIG	0	12 (16)	0	7 (27)	0	5 (10)	0.09
Glucocorticoid therapy with the 1st IVIG therapy	0	10 (13)	0	6 (23)	0	4 (8)	0.08
Glucocorticoid therapy with the 2nd IVIG therapy	0	1 (1)	0	0	0	1 (2)	1
Baseline CAAs	0	22 (29)	0	22 (85)	0	0	
One-month follow-up CAAs	3	10 (13)	0	8 (31)	3	2 (4)	< 0.01
Two-month follow-up CAAs	0	8 (10)	0	7 (27)	0	1 (2)	< 0.01
Six-month follow-up CAAs	0	4 (5)	0		0	1 (2)	0.1

CAAs: coronary artery aneurysms

IQR: interquartile range

IVIG: intravenous immune globulin

MD: missing data

Overall, 26 (34%) patients had a baseline Z-score ≥ 2, and 22 (29%) had a baseline Z-score ≥ 2.5. Ten (13%) patients had at least one CAA at the one-month follow-up, 8 (10%) at the two-month follow-up, and 4 (5%) at the six-month follow-up. Five (6%) patients had a recent (< 8 weeks) or concurrent SARS-CoV-2 infection. Patients with a baseline Z-score ≥ 2 were younger than patients with a normal baseline Z-score (median age 14 vs. 34 months respectively, *p*-value < 0.01). We did not find any other difference between the two groups. Table 3 summarizes the results of the risk scores.

**Table 3:** Results of risk scores. Data are presented in number (percentage). Table 1 describes the scoring systems. Harada score was created to predict the need for IVIG therapy in the Japanese population, Egami, Sano, and Kobayashi scores to predict IVIG resistance in the Japanese population, Kawanet and Kawanet-echo scores to predict IVIG resistance in the European population and Son score to predict CAA risk in the United States population

Score	Number of measurements	Low-Risk		High-Risk	
*Harada*	43	9 (21)		34 (79)	
*Egami*	70	15 (21)		55 (79)	
*Sano*	34	30 (88)		4 (12)	
*Kobayashi*	36	3 (8)		33 (92)	
*Kawanet*	54	38 (70)		16 (30)	
*Kawanet-echo*	54	51 (94)		3 (6)	
		**Low-Risk**	**Moderate-Risk**		**High-Risk**
*Son*	77	49 (64)	14 (18)		14 (18)

A baseline Z-score ≥ 2 was associated with CAA development at one month (OR 10, CI 2–72, *p*-value < 0.01 using the univariate analysis and OR 6, CI 1.2–42, *p*-value = 0.04 using the multivariate analysis) and two months (OR 18, CI 3-357, *p*-value < 0.01 univariate, OR 11, CI 2-226, *p*-value = 0.03 multivariate) but not at six-month follow-up. A baseline Z-score ≥ 2.5 showed a positive association with aneurysm development at one month (OR 8, CI 2–39, *p*-value < 0.01 univariate, OR 6, CI 1.3–33, *p*-value = 0.03 multivariate), two months (OR 10, CI 2–73, *p*-value < 0.01 univariate, OR 7, CI 1.4–55, *p*-value = 0.03 multivariate), but not at six months (OR 3, CI 0.3–23, *p*-value = 0.3). Table 4 gives the sensitivity and specificity of the baseline Z-score ≥ 2 and ≥ 2.5 to predict CAAs at one, two and six-month follow-up.

**Table 4:** Sensitivity and specificity of a baseline Z-score ≥ 2 and ≥ 2.5 to predict coronary artery aneurysms at one, two and six-month follow-up

	One-month CAAs		Two-months CAAs		Six-month CAAs	
	Sn	Sp	Sn	Sp	Sn	Sp
*Baseline Z-score ≥ 2*	80	72	87	72	75	68
*Baseline Z-score ≥ 2.5*	70	77	75	77	50	73

CAAs: coronary artery aneurysms

Sn: sensitivity

Sp: specificity

The Son score showed a positive association with CAAs at one month (OR 3, CI 1.3-7, *p*-value = 0.01), two months (OR 3, CI 1.3-8, *p*-value = 0.01), but not at six months (OR 2, CI 0.6-7, *p*-value 0.2). After adjusting for age and corticosteroid therapy, we did not find an association between the Son and CAAs during the follow-up period (OR 2, CI 0.8-5, *p*-value 0.2 at one month, OR 2, CI 0.8-6, *p*-value 0.1 at two months and OR 1.3, CI 0.3-5, *p*-value 0.7 at six months).

None of the other risk scores were associated with CAA development at one, two, and six-month follow-ups. Table 5 shows their ORs and *p*-values.

**Table 5:** Odds ratios and 95% confidence intervals between risk scores and baseline Z-score ≥ 2 and ≥ 2.5 with coronary artery aneurysms at one, two, and six month follow up

	One-month CAAs			Two-months CAAs			Six-month CAAs		
	*OR*	*CI*	*p-value*	*OR*	*CI*	*p-value*	*OR*	*CI*	*p-value*
*Baseline Z-score ≥ 2*	10	2–72	< 0.01	18	3-357	< 0.01	6	0.8–136	0.1
*Baseline Z-score ≥ 2.5*	8	2–39	< 0.01	10	2–73	< 0.01	3	0.3–23	0.3
*Harada*	0.3	0.1-2	0.2	0.6	0.1-5	0.6	0.2	0–2	0.2
*Egami*	1	0.3-8	0.8	0.4	0.1-2	0.2	0.2	0–2	0.2
*Sano*	0	NA-2e + 123	1	0	NA-6e + 186	1	0	NA-6e + 226	1
*Kobayashi*	0.2	0–5	0.3	1e + 185	0-NA	1	7e + 06	0-NA	1
*Kawanet*	0.2	0-1.4	0.2	0.4	0–2	0.4	0	NA-1e + 132	1
*Kawanet-echo*	3	0.1–30	0.5	4	0.2–46	0.3	0	NA-3e + 121	1
*Son*	3	1.3-7	0.01	3	1.3-8	0.01	2	0.6-7	0.2

CAAs: coronary artery aneurysms

CI: 95% confidence interval

NA: not applicable

OR: odds ratio

## Discussion

To our knowledge, this is the first study to evaluate the association between risk scores and baseline coronary Z-scores with CAAs and coronary dilations in Italy. We found a strong association of initial Z-score ≥ 2 and ≥ 2.5 with CAAs at one and two months of illness, with higher ORs of the baseline Z-score ≥ 2. These findings are consistent with the study conducted by Son et all in North America that reported a high predictive utility of a baseline Z-score ≥ 2 for CAA development [10]. Among the scores we tested, only the Son score showed a high predictive value for CAAs at one and two months of illness [11]. In our sample, a Z-score ≥ 2 showed a stronger association with CAAs at one and two-month follow-ups. We also extended our follow-up until six months, while Son et all evaluated only the presence of CAAs at 4 and 8 weeks of illness [10, 11]. The baseline Z-scores were not significantly associated with CAAs at 6 months, but this result could be affected by the low number of patients who presented CAAs at 6 months, which limits the power of the study.

We tested both a baseline Z-score ≥ 2 and ≥ 2.5, with a Z-score ≥ 2 showing higher associations with the outcomes. We also evaluated the Kawanet and the Kawanet-echo score performances, which were developed in the European cohort [12, 13], but none of them was associated with CAAs. Consisting with previous studies, we found that Japanese scores have low performance for the non-Japanese population [9, 10, 12, 18, 19].

The European guidelines recommend that all KD patients should undergo echocardiography at baseline, then after 2 weeks after IVIG administration, and then after 6–8 weeks after disease onset [14]. The Italian guidelines for KD recommend an echocardiogram on all patients when diagnosed with KD, then at 2, 4, and 8 weeks for uncomplicated cases and assessing the Z-score of the coronary artery at every stage [15]. If echocardiography is not available the IVIG infusion should not be delayed [14, 15].

Our findings suggest that patients with a baseline Z-score ≥ 2 have a higher risk for CAAs in the long term and that in our contest the baseline Z-score is the best predictor for CAAs development. Since the CAA is the most serious complication in KD [20], we encourage that multi-centric European studies be performed in this subgroup of patients to test more aggressive treatment from the diagnosis.

We found that 34% of our patients had baseline Z-score ≥ 2 and 29% had a baseline CAA. Our incidence of coronary artery dilatation and aneurysm is higher than the one found by Son et al. (29% and 19% respectively) [11] but lower than the one found by Dallaire et al. (65% and 33.5% respectively) [21]. The incidence of coronary artery dilation in different ethnic groups is not established and, in accord with the AHA, it could be more common than previously thought [16].

SARS-CoV-2 seems to trigger both KD and multisystem inflammatory syndrome (MIS-C) phenotypes [22]. We did not include MIS-C patients according to the World Health Organization and Centers for Disease Control and Prevention Guidelines who did not meet KD diagnostic criteria [23, 24]. Differential diagnosis between KD and MIS-C could be difficult because they share overlapping clinical presentations [25, 26]. All the patients included in the present study were discharged with a diagnosis of KD, moreover, four expert clinicians, two paediatric immunologists and two paediatric cardiologists, analysed the cases to include to better discriminate between the two diagnoses. A Korean study did not find differences in cardiac complications between patients with KD with and without a recent COVID-19 infection [27]. Since in our sample, only five patients had recent or concurrent SARS-CoV-2 infection, we did not evaluate the differences between the two groups. Larger studies should evaluate the difference in cardiac complications in European cohorts.

We had a variation in echocardiogram timing. The indications regarding corticosteroid therapy changed during the observational period [14], which could have resulted in different therapeutic management of our patients. We tried to reduce the bias of this possible confounding factor by adjusting our analyses for corticosteroid therapy. Moreover, we could not calculate the score value for many patients, such as Sano and Kobayashi scores which were calculated for less than half of the sample. Despite the European and Italian recommendations, many centres cannot perform an echocardiogram before the initiation of the treatment [28]. Even if relatively large, our dataset could not allow us to assess the association between the scores and baseline Z-score with CAAs at six-month follow-up, because only 4 patients had CAAs at 6 months.

We tested seven scores, some of them recently proposed, anyway, we did not test the recently developed score in the Chinese population from Lui et all [29], whose performance should be assessed in the non-Asian population. However, the Lui score does not contain the baseline Z-score as a variable and our results suggest that it is not the best scoring system in our contest.

The strengths of our research are the introduction of the six-month follow-up outcome that, even if the limited sample size could not allow us to make a firm conclusion, shows a result changing from the two-month follow-up, suggesting that future studies should extend the observational period. We uniformly assessed the coronary artery Z-score in referring to Dallaire and Dahdah Z-scores [17]. Moreover, the monocentric setting allows a more uniform coronary Z-scores measurement.

## Conclusion

Patients with a baseline Z-score ≥ 2 are at higher risk for CAAs in the long term, suggesting that more aggressive treatment should be tested with ad hoc studies. Even if inferior to the baseline Z-score alone, the Son score was associated with CAAs at 4 and 8 weeks from disease onset, it should be evaluated in larger European samples. Moreover, predictors for CAAs outcome should be considered in a longer observational period than 8 weeks from disease onset, since we found changes in our results between the two- and six-month follow-up.

## Data Availability

Data are available at the corresponding author upon reasonable request.

## Abbreviations

AHA: American Heart Association
ASA: Acetylsalicylic acid
CAA: Coronary artery aneurysm
CI: Confidence interval
IVIG: Intravenous immune globulin
KD: Kawasaki disease
MIS-C: Multisystem inflammatory syndrome
OR: Odds ratio
